# The experiences of minority language users in health and social care research: A systematic review

**DOI:** 10.1002/hpm.3825

**Published:** 2024-08-26

**Authors:** Llinos Haf Spencer, Beryl Ann Cooledge, Zoe Hoare

**Affiliations:** ^1^ Welsh Institute for Health and Social Care University of South Wales Pontypridd UK; ^2^ Language Awareness Infrastructure Support Service (LLAIS) North Wales Organisation for Randomised Trials in Health and Social Care (NWORTH) Bangor University Bangor UK; ^3^ North Wales Organisation for Randomised Trials in Health and Social Care (NWORTH) Bangor University Bangor UK

**Keywords:** health research, minority language, speakers, trials, users

## Abstract

**Background:**

The planning and management of health policy is directly linked to evidence‐based research. To obtain the most rigorous results in research it is important to have a representative sample. However, ethnic minorities are often not accounted for in research. Migration, equality, and diversity issues are important priorities which need to be considered by researchers. The aim of this systematic review (SR) is to explore the literature examining the experiences of minority language users in Health and Social Care Research (HSCR).

**Method:**

A SR of the literature was conducted. SPIDER framework and Cochrane principles were utilised to conduct the review. Five databases were searched, yielding 5311 papers initially. A SR protocol was developed and published in PROSPERO: https://www.crd.york.ac.uk/prospero/display_record.php?ID=CRD42020225114analysis.

**Results:**

Following the title and abstract review by two reviewers, 74 papers were included, and a narrative account was provided. Six themes were identified: 1. Disparities in healthcare; 2. Maternal health; 3. Mental health; 4. Methodology in health research; 5. Migrant and minority healthcare; 6. Racial and ethnic gaps in healthcare. Results showed that language barriers (including language proficiency) and cultural barriers still exist in terms of recruitment, possibly effecting the validity of the results. Several papers acknowledged language barriers but did not act to reduce them.

**Conclusion:**

Despite research highlighting cultures over the past 40 years, there is a need for this to be acknowledged and embedded in the research process. We propose that future research should include details of languages spoken so readers can understand the sample composition to be able to interpret the results in the best way, recognising the significance of culture and language. If language is not considered as a significant aspect of research, the findings of the research cannot be rigorous and therefore the validity is compromised.

## Highlights

- Despite health and social care research acknowledging different cultures over the past 40 years, this needs to be fully embedded into the research process.
- Ethnic background and languages spoken by the research participants should be identified and addressed throughout the research process.
- Language and cultural preferences should be appropriately included in the analysis.
- Future research should include details of ethnicity and languages spoken so readers can understand the sample composition to interpret the results in the best way.

## 1 INTRODUCTION

### 1.1 Ethnic minorities in health research overview

Ethnic minorities across the globe encounter disparities in healthcare.^1^, ^2^ The completeness of ethnicity data within healthcare has historically been poor.^3^ However, the gap between knowledge translation and minority‐language speakers is now starting to close with new legislation^4^ and interest by researchers.^5^

For research to improve the health of our communities it needs to serve the interests of all, recognising diversity and acknowledging the importance of culture. Achieving this needs a systematic approach, starting with asking the right questions, designing inclusive trials, removing regulatory, financial, and institutional barriers to inclusion, concluding with building long‐term relationships with under‐served groups. Crucially, we need to ensure that research is designed so that its participants reflect those who might benefit from the results.^6^ Treweek et al., (2021) noted: ‘Thinking about the number of people in our trials is not enough: we need to start thinking more carefully about who our participants are’. (Page 10 of 12 pages).

In 2013, a call for action was made to include ethnic minorities in research,^7^ and by 2021 the framework for including ethnic minorities in health research was developed.^6^ There is increasing evidence to suggest that culture and language‐responsive research enhances rigour, inclusivity, and fairness. Engaging with research participants through a language that is meaningful to them is key to good clinical research practice.^8^, ^9^ However, research from around the world, suggests that minority language speakers are under‐represented in health research.^7^ There are varying reasons for this, including issues regarding recruitment, especially when the researcher does not share the same culture.

Pyett (2002) noted:

It maybe difficult,… for a researcher to establish credibility with a marginalised or minority group unless they are a member of that group. Many conventional research techniques are not appropriate for these groups since language, literacy and cultural difference can lead to misunderstandings and mis‐interpretations. (page 332).^10^

The Welsh Government also has policies for an ‘inclusive Wales’^11^ in which all people from all backgrounds, abilities and religions are encouraged to take an active part within in all aspects of community life. The Prosperity for Wales National strategy^11^ highlight the following:

Communities prosper where people can participate fully and play an active role in shaping their local environment, influencing the decisions which affect them. P. 19

Taking part in research is not high on the agenda of the typical member of the public, and seems to be even less so for minority language speakers.^12^ In Wales, according to the 2011 Census, 19% (562,000) of usual residents in Wales aged three and over reported they could speak Welsh. Thirty per cent (169,000) of this group were aged between three and 15 years old.^13^ There is currently no evidence to suggest Welsh speakers do or do not take part in research as the information is not routinely collected as part of the demographic data collection in a health research project or clinical trial. This suggests cultural sensitivity and language awareness cannot be assumed, and therefore must be measured. In his Theory of Durable Inequality^14^ Tilly argues that the clumping together of ethnic categories with socio‐economic categories helps to reinforce exploitation. This could lead to durable inequalities.^15^ The under‐serving of ethnic minorities in HSCR is an issue which needs more exploration and explanation especially during this time of increased global mobility and migration.^2^ The aim of this systematic review (SR) was to investigate the issues surrounding minority language speakers in HSCR.

## 2 MATERIALS AND METHODS

A SR of the literature was utilised based on a step by step approach.^16^ Five databases were searched including the following: Applied Social Science Index (ASSIA), CINAHL Plus with Full text, PsycINFO, PubMed and Web of Science (Core Collection). Grey literature was also be sought through the Google Search Engine and reference sections from other papers. The searches were restricted to English, from January 2000 to December 2020. A 20‐year time was selected since minority language barriers in research began to be reported around the early 2000s.

The search strategy was developed in conjunction with an information scientist at Bangor University, and consists of five levels: population, phenomenon of interest, design, evaluation, and research type, according to the SPIDER framework. The search strategy information is shown in Appendix 1. As we were only interested in patient experience of taking part in studies as minority language users, we are not comparing minority language users with the main linguistic group of their country, therefore there is no control or comparator in this SR approach. The included papers were quality appraised using the appropriate appraisal checklists (See Supporting Information S1). Ethical approval was not necessary for this SR.

The 74 included studies were a mixture of qualitative studies (*n* = 22), quantitative studies (*n* = 10 including *n* = 1 cohort study and *n* = 9 survey studies including on‐line and pilot surveys), mixed methods studies (*n* = 9) cross‐sectional studies (*n* = 5), case report (*n* = 1); and reviews (*n* = 27). See Appendix 2.

## 3 RESULTS

**FIGURE 1:**
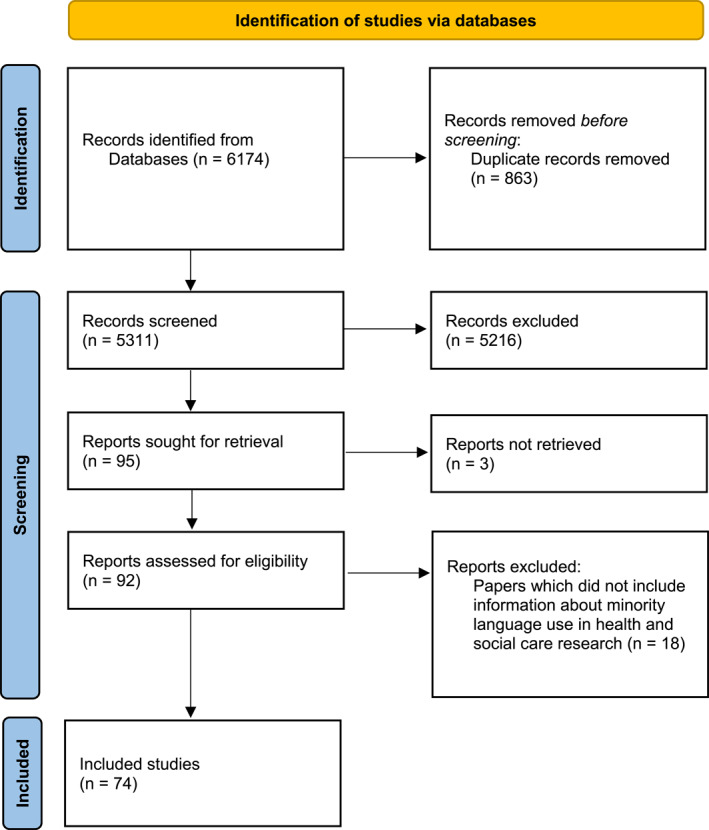
Identification of studies via databases. Page MJ, McKenzie JE, Bossuyt PM, Boutron I, Hoffmann TC, Mulrow CD, et al. The PRISMA 2020 statement: an updated guideline for reporting systematic reviews. BMJ 2021; 372: n71. 10.1136/bmj.n71 (Page et al., 2021).

The Preferred Reporting Items for Systematic Reviews and Meta‐Analyses (PRISMA)^17^ diagram is shown in Figure 1 and the list of studies is included in Table 1. An in‐depth critical review of the quality of each article was conducted (see Supporting Information S1) and data extracted according to study design (Appendix 2). A narrative synthesis of the findings will be presented in this analysis section.

**TABLE 1:** List of papers by author, country, date and domain of interest.

#	Author(s)	Country	Year	Domain of interest	Study participants	Study design
1.	Hunt and Bhopal (Hunt & Bhopal, 2004)	Scotland, UK	2004	Disparities in health care research.	This was a methodological commentary regarding including non‐English speakers in research.	Research synthesis
2.	Gibbs et al. (Gibbs, Nsiah‐Jefferson, McHugh, Trivedi, & Prothrow‐Stith, 2006)	USA	2006	Disparities in health care research.	*N* = 4 policies from the USA were analysed for this review.	Research synthesis
3.	Jacobs et al. (Jacobs et al., 2006)	USA	2006	Disparities in health care research.	*N* = 2 systematic reviews of the literature.	Research synthesis
4.	Sue and Dhindsa (Sue & Dhindsa, 2006)	USA	2006	Disparities in health care research.	This was a review addressing ethnic and racial health and health care disparities research.	Research synthesis
5.	Beresford (Beresford, 2007)	England, UK	2007	Disparities in health care research.	This was a descriptive narrative to develop new directions in health inequalities research.	Research synthesis
6.	Lau et al. (Lau, Chang, & Okazaki, 2010)	USA	2010	Disparities in health care research.	This was a commentary on randomised controlled trials looking at specific challenges facing investigators conducting ethnically inclusive trials.	Research synthesis
7.	Goode et al. (Goode, Carter‐Pokras, Horner‐Johnson, & Yee, 2014)	USA	2014	Disparities in health care research.	This was a commentary on the lack of existing literature which focuses on the intersection of race, ethnicity, and disability.	Research synthesis
8.	Kaplan (Kaplan, 2014)	USA	2014	Disparities in health care research.	This was a commentary on the quality of data on ‘race’ and ‘ethnicity’ in the USA.	Research synthesis
9.	Coustasse et al. (Coustasse, Bae, Arvidson, & Singh, 2010)	USA	2010	Disparities in health care use	*N* = 31,875	Survey study
10.	Wilk et al. (Wilk, Maltby, & Phillips, 2018)	Canada	2018	Disparities in health care use.	From the 2006 aboriginal peoples surveys (APS) survey, 20,720 respondents were included and 24,150 from the 2012 APS.	Cross‐sectional study
11.	Chirewa, 2012 (Chirewa, 2012)	UK	2012	Disparities in health care use.	The research participants were drawn from the national government organisations (NGO's) forum affiliates (*n* = 30), NHS primary care trusts (PCTs) (*n* = 2) and an expert group (academics) (*N* = 4).	Mixed method study
12.	Gaston‐Johansson et al. (Gaston‐Johansson, Hill‐Briggs, Oguntomilade, Bradley, & Mason, 2008)	USA	2008	Disparities in health care use.	*N* = 9 participants in each of the 6 focus groups. (*N* = 42 in total)	Qualitative study
13.	Akhavan and Karlsen (Akhavan & Karlsen, 2013)	Sweden	2013	Disparities in health care use.	*N* = 5 × ‘migrant’ health service clients and 5 × physicians.	Qualitative study
14.	Hahm et al. (Hahm, Lahiff, Barreto, & Chen, 2008)	USA	2008	Disparities in health care use.	*N* = 2230 ethnic minority adolescents were interviewed over the telephone.	Survey study
15.	Di Pietro & Illes (Di Pietro & Illes, 2014)	Canada	2014	Disparities in health care use.	*N* = 52 reports published since 1981 were included in this systematic review.	Systematic review
16.	Strohschein et al. (Strohschein, Merry, Thomas, & Gagnon, 2010)	Canada	2010	Maternal health	For the PACBIRTH study, 5 refugee/asylum seeking monolingual women per language, speaking Hindi, Tamil, Urdu, Spanish, and French participated in the testing. For the KAP study, 3 monolingual French‐speaking and refugee/asylum‐seeking women participated, as well as 10 refugee/asylum‐seeking monolingual Urdu, Tamil, and Hindi‐speaking couples. Monolingual participants came from Mexico, Peru, Colombia, Pakistan, Sri Lanka, India, Cameroon, and the Congo	Qualitative study
17.	Johnsen et al. (Johnsen et al., 2020)	Denmark	2020	Maternal health	*N* = 18 midwives	Qualitative study
18.	Huang et al. (Huang et al., 2019)	China	2019	Maternal health	*N* = 10 qualitative papers were included in this systematic review	Systematic review
19.	Durbin et al. (Durbin, Sirotich, & Durbin, 2017)	Canada	2017	Mental health	*N* = 1449 mental health services clients.	Cross‐sectional study
20.	Sadavoy et al. (Sadavoy, Meier, & Ong, 2004)	Canada	2004	Mental health	*N* = 10 in Chinese speaking focus groups *N* = 7 in Tamil speaking focus groups	Qualitative study
21.	Shattell et al.s (Shattell, Hamilton, Starr, Jenkins, & Hinderliter, 2008)	USA	2008	Mental health	*N* = 7 community members (2 × male and 5 × female) *N* = 1 health educator *N* = 1 doctoral student in nursing *N* = 2 undergraduate nursing students *N* = 1 principal investigator	Qualitative study
22.	De La Torre (De La Torre, 2009)	USA	2009	Mental health	*N* = 20 Hispanic adults of both sexes (10 males and 10 females)	Qualitative study
23.	Rose and Cheung (Rose & Cheung, 2012)	USA	2012	Mental health	*N* = 54 articles published between 2001 and 2011.	Research synthesis
24.	Kim et al. (Kim et al., 2011)	USA	2011	Mental health	*N* = 372 in total (Latino, Hispanic and Asian adults).	Survey study
25.	Brisset et al. (Brisset et al., 2014)	Canada	2014	Mental health	*N* = 113 primary care practitioners in Montreal.	Survey study
26.	Lu et al. (Lu, Dear, Johnston, Wootton, & Titov, 2014)	Australia	2014	Mental health	*N* = 1449 mental health services clients	Survey study
27.	Woodall et al. (Woodall, Morgan, Sloan, & Howard, 2010)	England, UK	2010	Mental health	*N* = 49 papers were included in this systematic review.	Systematic review
28.	Brown et al. (Brown, Marshall, Bower, Woodham, & Waheed, 2014)	UK	2014	Mental health	*N* = 9 papers included in the systematic review.	Systematic review
29.	Tadić et al. (Tadić et al., 2010)	UK	2010	Methodology in health research	*N* = 32—interviewed (in stage 1) *N* = 44 (in stage 2).	Mixed method study
30.	Mowlabaccus and Jodheea‐Jutton (Mowlabaccus & Jodheea‐Jutton, 2020)	Mauritius	2020	Methodology in health research	Qualitative: *N* = 23 open‐ended question on‐line survey. Survey: *N* = 350 completed questionnaires	Mixed method study
31.	Dingoyan et al. (Dingoyan, Schulz, & Mosko, 2012)	Germany	2012	Methodology in health research	The number of participants varied between 7 and 12 individuals per focus group.	Qualitative study
32.	Schildmann et al. (Schildmann et al., 2016)	Germany and England	2016	Methodology in health research	*N* = 15 German and *N* = 10 UK interviews were conducted.	Qualitative study
33.	Squires et al. (Squires et al., 2019)	USA	2019	Methodology in health research	*N* = 35 home health care providers.	Qualitative study
34.	Murray and Buller (Murray & Buller, 2007)	England, UK	2007	Methodology in health research	*N* = 207 ‘original research pieces’ published in the BMJ were discussed in this review.	Research synthesis
35.	Wells and Zebrack (Wells & Zebrack, 2007)	USA	2007	Methodology in health research	In this review previous research was not selected but the authors utilised a social‐ecological perspective to describe their findings.	Research synthesis
36.	Martinez (Martinez, Carter‐Pokras, & Brown, 2009)	USA	2009	Methodology in health research	This was a review describing the lessons learnt in using a participatory approach.	Research synthesis
37.	Yildiz and Bartlett (Yildiz & Bartlett, 2011)	England, UK	2011	Methodology in health research	*N* = 27 studies were included in this review.	Research synthesis
38.	Nishita and Browne (Nishita & Browne, 2013)	Hawaii, USA	2013	Methodology in health research	This was a broad literature review.	Research synthesis
39.	Waheed et al. (Waheed, Hughes‐Morley, Woodham, Allen, & Bower, 2015)	England, UK	2015	Methodology in health research	*N* = 9 studies were included.	Research synthesis
40.	Morville and Erlandsson (Morville & Erlandsson, 2016)	Sweden	2016	Methodology in health research	*N* = 21 articles were included in this literature review.	Research synthesis
41.	Premji et al. (Premji, Kosny, Yanar, & Begum, 2020)	Canada	2020	Methodology in health research	*N* = 6 studies by the author were included in this review.	Research synthesis
42.	Mosconi (Mosconi et al., 2016)	Italy	2016	Methodology in health research	*N* = 1852 survey responses.	Survey study
43.	MacFarlane et al. (MacFarlane, Singleton, & Green, 2009)	Ireland and England	2009	Migrant people's health care needs.	Ireland: *N* = 26 Serb Croat and Russian speaking refugees and asylum seekers (*n* = 16 females and *n* = 10 males). England: Focus groups (11 focus groups; *n* = 61 participants) Semi‐structured interviews (*n* = 28 participants).	Qualitative study
44.	Doyle et al. (Doyle, Rager, Bates, & Cooper, 2013)	USA	2013	Migrant people's health care needs.	*N* = 9 healthcare providers *N* = 11 social service providers *N* = 20 migrant and seasonal farm workers (*N* = 40 in total)	Qualitative study
45.	Betancourt et al. (Betancourt, Frounfelker, Mishra, Hussein, & Falzarano, 2015)	USA	2015	Migrant people's health care needs.	Free list group *N* = 39 = Somali Bantu *N* = 62 = Bhutanese refugees Key Informant group *N* = 21 = Somali Bantu *N* = 40 = Bhutanese refugees	Qualitative study
46.	Hunter‐Adams and Rother (Hunter‐Adams & Rother, 2017)	South Africa	2017	Migrant people's health care needs.	Semi‐structured interviews: Congolese (*n* = 7) Somali (*n* = 8) Zimbabwean (*n* = 8) women living in Cape Town *N* = 9 focus groups including men and women.	Qualitative study
47.	Chowdhury (Chowdhury, Naeem, Ferdous, Chowdhury, & Goopy, 2021)	Canada	2021	Migrant people's health care needs.	*N* = 31 studies were included in this systematic review.	Systematic review
48.	Kale & Syed (Kale & Syed, 2010)	Norway	2010	Minority participation in health care	*N* = 453 participants from both primary and specialised healthcare facilities.	Cross‐sectional study
49.	Angus et al. (Angus et al., 2013)	Canada	2013	Minority participation in health care	*N* = 35 qualitative studies were included in this meta‐ analysis.	Meta analysis
50.	Wang and Kwak (L. Wang & Kwak, 2015)	Canada	2015	Minority participation in health care	*N* = 8 focus groups (*n* = 54 in total; 81.5% female and 18.5% male).	Mixed method study
51.	Wang (A. Wang et al., 2019)	Canada	2019	Minority participation in health care	Quantitative chart review: *n* = 2420 registered charts Qualitative focus groups: *n* = 13 participants	Mixed method study
52.	Claydon‐Platt et al. (Claydon‐Platt, Manias, & Dunning, 2013)	Australia	2013	Minority participation in health care	*N* = 11 people with diabetes, *N* = 10 carers and *N* = 10 health professionals were interviewed. (*N* = 31 in total)	Qualitative study
53.	Tatari et al. (Tatari et al., 2020)	Denmark	2020	Minority participation in health care	*N* = 37 women from 10 different non‐Western countries participated in the study.	Qualitative study
54.	Castillo (Castillo, Gandy, Bradko, & Castillo, 2019)	USA	2019	Minority participation in health care	*N* = 18 papers included in the systematic review.	Systematic review
55.	Blanchet et al. (Blanchet et al., 2017)	Canada	2017	Minority participation in health care research	*N* = 259 parent and child dyads (251 biological mothers, one adoptive mother, three fathers).	Cohort study
56.	Eriksson‐ Sjöö et al. (Eriksson‐Sjöö, Cederberg, Östman, & Ekblad, 2012)	Sweden	2012	Minority participation in health care research	*N* = 78 newly‐arrived Arabic‐speaking adult refugees in Malmö, Sweden.	Mixed method study
57.	Ahlmark et al. (Ahlmark, Algren, Holmberg, & Norredam, 2014)	Denmark	2014	Minority participation in health care research	*N* = 177,639 (survey). Also data from 10 immigrants and 13 descendants between the ages of 18–54.	Mixed method study
58.	Robinson and Trochim (Robinson & Trochim, 2007)	USA	2007	Minority participation in health care research	*N* = 20 steering committee members (*n* = 20), *N* = 16 community advisory board members regional *N N* = 6 advisory board members *N* = 5 lay community members (*n* = 5)	Qualitative study
59.	Fisher (Fisher, 2011)	Australia	2011	Minority participation in health care research	*N* = 54 from five communities in Australia. *N* = 24 from health and support agency staff who provide services to them. Agency support staff represented a range of professional perspectives. (*N* = 78 in total)	Qualitative study
60.	French and Stavropoulou (French & Stavropoulou, 2016)	UK	2016	Minority participation in health care research	*N* = 12 specialist nurses representing 7 different clinical specialities and 7 different NHS trusts.	Qualitative study
61.	O’Connor et al. (O’Connor, Adem, & Starks, 2018)	USA	2018	Minority participation in health care research	Community leader interviews (*n* = 6) and focus groups with lay members (*n* = 16) from the three largest East African communities in the Seattle area (Eritrean, Ethiopian and Somali)	Qualitative study
62.	Greene et al. (Greene, Karavatas, Cooper, & Zamorano‐Torres, 2013)	USA	2013	Minority participation in health care research	*N* = 30 patients from the Washington, DC metropolitan area, whose primary language is Spanish	Survey study
63.	Carlini et al. (Carlini, Safioti, Rue, & Miles, 2015)	USA	2015	Minority participation in health care research	Brazilian community members living in the USA: (Florida, California and New Jersey).	Survey study
64.	Falla et al. (Falla, Veldhuijzen, Ahmad, Levi, & Richardus, 2017)	The Netherlands	2017	Minority participation in health care research	*N* = 238 respondents to on‐line survey.	Survey study
65.	Du (Du, 2018)	USA	2018	Providing health care services to ethnic minority patients.	*N* = 1. This case report was a reflection of a graduate medical student who provided real‐life examples regarding language and communication.	Case report
66.	Peek et al. (Peek et al., 2012)	USA	2012	Providing health care services to ethnic minority patients.	*N* = 167 physician organisations (of differing levels of membership).	Cross‐sectional study
67.	Silveira et al. (Silveira et al., 2020)	USA	2020	Providing health care services to ethnic minority patients.	*N* = −16,415 Hispanic/Latino adults in the U.S.	Cross‐sectional study
68.	Bhuiyan et al. (Bhuiyan, Urmi, Chowdhury, & Rahman, 2019)	Bangladesh	2019	Providing health care services to ethnic minority patients.	*N* = 50 participants of different age groups. *N* = 44 male *N* = 6 female	Mixed method study
69.	Tan and Denson (Tan & Denson, 2019)	Australia	2019	Providing health care services to ethnic minority patients.	*N* = 38 bilingual/multilingual psychologists working in Australia in 2015. *N* = 11 participants undertook supplementary telephone interviews	Mixed method study
70.	Vandan et al. (Vandan et al., 2020)	Hong Kong, China	2020	Providing health care services to ethnic minority patients.	*N* = 22 health care professionals	Qualitative study
71.	Schwei et al. (Schwei, Del Pozo, Agger‐Gupta, Alvarado‐Little, Bagchi, Hm Chen, et al., 2016)	USA and Canada	2016	Providing health care services to ethnic minority patients.	*N* = 136 studies prior to 2003 and *N* = 426 studies from 2003 to 2010.	Research synthesis
72.	Joo and Liu (Joo & Liu, 2020)	South Korea	2020	Providing health care services to ethnic minority patients.	*N* = 8 papers were included in this systematic review.	Systematic review
73.	Clarke et al. (Clarke et al., 2013)	USA	2013	Racial and ethnic gaps in health care.	*N* = 11 systematic reviews were included in this meta analysis.	Meta analysis
74.	Haley et al. (Haley, Southwick, Parikh, Farrar‐Edwards, & Boden‐Albala, 2017)	USA	2017	Racial and ethnic gaps in health research.	*N* = 29 clinical research coordinators (CRCs)	Qualitative study

For this narrative analysis the 74 included papers were categorised according to the following six themes, based on their main findings:

1. Disparities in healthcare
2. Maternal health
3. Mental health
4. Methodology in health research
5. Migrant and minority healthcare
6. Racial and ethnic gaps in healthcare

Further sub‐themes were identified within the six main themes (see Table 2).

**TABLE 2:** Themes and sub‐themes for minority language speakers in health research.

Theme	Sub‐theme	Number of papers
1. Disparities in health care	Disparities in health care research	8
	Disparities in health care use	7
2. Maternal health		3
3. Mental health		10
4. Methodology in health research		14
5. Migrant and minorities in health care	Migrant people's health care needs	5
	Minority participation in health care	7
	Minority participation in health care research	10
	Providing health care services to ethnic minority patients	8
6. Racial and ethnic gaps	Racial and ethnic gaps in health care	1
	Racial and ethnic gaps in health research	1
	**Total**	**74**

*Note*: The number in bold is the total number of papers.

### 3.1 Theme 1: Disparities in healthcare

Disparities in healthcare research (*n* = 8 papers) and disparities in healthcare use (*n* = 7 papers) were the two sub‐themes. All these papers (*n* = 7) were research syntheses focussing on the need for the research community to assists healthcare providers and policymakers with the evidence they require to design and effectively implement linguistically accessible services to limited English proficiency (LEP) patients.^12^ Even when people belonging to another culture speak fluent English they do not necessarily share the beliefs and values of native English speakers.^18^ Authors also highlighted that research in both disability and ethnicity frequently fails to address the multiple cultural identities within population groups.^19^

There were seven disparities in healthcare use papers.^20^, ^21^, ^22^, ^23^, ^24^, ^25^, ^26^ A SR highlighted the paucity of research conducted with Aboriginal groups in Canada.^25^ The inclusion of ethnic minority groups should be improved to ensure the views of minority groups such as the Indian, Inuit and Métis peoples are represented in research planning and decision‐making, from idea conception and design of projects through to the analysis and dissemination of study results. Survey studies^20^, ^24^ have also acknowledged the language used in a survey may affect respondents' self‐reported health status.^20^ There is also recognition that language spoken is an increasing issue in healthcare research and delivery in the USA due to in‐migration from many countries including South American countries, China, Vietnam, Korea and Cambodia.^24^ The qualitative studies^23^, ^26^ emphasise the point that the power disparity between doctors and ‘migrant’ patients encourages a sense of powerlessness and mistreatment among patients.^23^ Authors have also noted that more ethnic minority speakers and bilingual providers should be trained to provide a good health service for minority language speakers.^26^ Other authors have noted that embracing the different cultures of stakeholders and partner organisations is imperative for successful participatory action research.^22^

### 3.2 Theme 2: Maternal health

This theme included three papers which specifically investigated maternal health in relation to minority populations.^27^, ^28^, ^29^ In the SR eight studies out of 10 did not report the preferred language of the individuals.^29^ A Canadian qualitative study included linguistically validating a maternal health measure into several different languages including Hindi, Tamil, Urdu, Spanish, and French and highlighted the need to be culturally as well as linguistically aware.^27^ In a maternal mental health study to explore the feasibility of an intervention, the authors noted that language proficiency was of great importance for the provision of care and communication difficulties caused adverse events. Even though the hospital offered interpreter assistance, interpreters were not always available. Sometimes, immigrant women would bring their partner, a relative or a friend to interpret for them. This was described as potentially problematic due to the lack of confidentiality and the lack of ability to assess the quality of the translation.^28^

### 3.3 Theme 3: Mental health

This theme included studies (*n* = 10) which specifically investigated mental health in relation to minority populations. A SR investigating barriers to taking part in mental health research with particular reference to gender, age, and ethnicity included 49 papers.^30^ Strategies to overcome barriers to taking part in health research included utilising bilingual staff to recruit, and avoiding the use of stigmatising language in marketing material. Similarly another SR noted the importance of considering barriers and find solutions to overcome obstacles from the start of the study.^31^ A further study discussed the inadequacies of the Diagnostic and Statistical Manual 5 (DSM‐5) health classification system resulting in limited diagnoses of people of colour and mistrust leading to inadequate treatment.^32^

The dearth of appropriate psychiatrists with language and cultural competency is the most clearly identified gap in mental health service provision in Canada.^33^ A qualitative study also used focus groups to investigate the mental health service needs of a Latino population in 2006–2007 and noted that the most effective means of building relationships with Latino clients was to have the same culture as well as speak the same language.^34^ Similarly a study indicated that clients with language concordant providers have better outcomes across all nine need domains including housing, food, transportation, and community living skills (all *p* values < 0.02).^35^

Another qualitative study investigated the concern and needs of Hispanic patients having psychiatric outpatient treatment.^36^ Language barriers constituted an obstacle to treatment progress because it triggered poor relationships with the psychiatrist, poor quality of care, and even participants' early termination of treatment. Quotes included:

She does not speak Spanish. I would like someone that I could share my problems and feelings in my language. …I would like to have a real conversation… (P. 234)^36^

A 2011 survey examined the effect of LEP on mental health service use among immigrant adults with psychiatric disorders and found LEP was a barrier to mental health service use among Latino immigrants with psychiatric disorders.^37^ A survey was developed to address barriers in accessing mental healthcare.^38^ Having access to interpreters was considered as the most important resource to overcome language barriers, but the great majority of practitioners had not been trained to work with interpreters. Most interpreted consultations involved ad hoc interpreters (e.g., family or friends). This finding was consistent with the literature regarding ad hoc interpreters being able to offer immediate availability, continuity, and were trusted by clients. Disadvantages being that ad hoc interpreters did not necessarily convey clients' disagreement or resistance about the diagnostic process or treatment.^38^ Another survey found Chinese‐speaking students in Australia were at high‐risk for developing psychological distress, with common cultural barriers including language difficulties and not perceiving symptoms serious enough to warrant treatment.^39^

### 3.4 Theme 4: Methodology in health research

A review of language barriers in qualitative health research noted that there remain gaps and debates with respect to the relevant ethical and methodological guidance set forth by funding agencies.^40^ In Canada, the Tri‐Council Policy Statement or TCPS 2 (2014) on ethical conduct for research involving humans, notes that researchers shall not exclude individuals from participating in research based on attributes such as culture, language, religion, race, disability, sexual orientation, ethnicity, linguistic proficiency, gender, or age, unless there is a valid reason for the exclusion.^40^ A scoping study which assessed methodological challenges when doing research that includes ethnic minorities described the option of using bilingual interviewers from the same ethnic minority group, further highlighting the importance of using interviewers with cultural knowledge.^41^ Similar results regarding cultural awareness were found in a qualitative study focussing on people with Turkish migration backgrounds living in Germany.^42^

Another methodological issue was sending research invitations in only one language can result in poor recruitment. For example, in one study less than 50% of children and adolescents from ethnic minority backgrounds responded because the letters were in English only.^43^ Barriers relating to interpreters include long waiting times, the need to pay professional interpreters and accuracy of interpretation.^44^ Further methodological issues identified in this SR were distrust of the value of research by minority groups which could be reduced by ensuring that the informed consent is in the language of the participant.^45^ The European Communication on Research Awareness Need (ECRAN) have developed research materials and tools in different languages, which are freely available under a Creative Commons licence to help researchers to overcome methodological barriers.

### 3.5 Theme 5: Migrant and minorities in healthcare

This theme included papers regarding language barriers, the use of informal and formal interpreters, and barriers to research participation. A qualitative study found in both England and Ireland there is a need for more attention to the implementation of policies for language barriers.^46^ Example comments from service users included:

CARe Z3, who takes her daughter to her GP consultations to interpret said that she does not go to see the doctor if the complaint is of a personal nature that she does not want her daughter to be involved in.^46^

A mixed‐method study captured insights into the experience of Korean immigrants in seeking and receiving healthcare in Canada.^47^ Almost all the participants preferred to have a Korean‐speaking family physician:

I have lived here a long time, so my English is okay for basic things. But when my symptoms are very complicated …. I cannot express completely my symptoms to the doctors. That is something I have experienced hundreds of times. (P. 344)^47^

Another mixed method study was also conducted in Canada and focussed on breast and colorectal cancer screening barriers among immigrants and refugees.^48^ Interpreters are important, but time consuming and the forms and Faecal Occult Blood Test (FOBT) kits were only available in English or French.^48^

We have 30 minutes per appointment typically, but when they’re dealing with …..language line, interpretation services, you don’t necessarily have that much time to explain … the importance of screening (page 477).^48^

Another study concerning cancer screening was conducted in Denmark, with 37 women from 10 different non‐Western countries.^49^ Knowledge about cancer screening was fragmented, often due to inadequate Danish language skills and a general mistrust in the Danish healthcare system.

… So, all the material you get you don’t read it because you don’t understand it … If it is only in Danish, no one looks at it. (Participant) (P. 6 of 10).^49^

Similarly, researchers in Texas interviewed seasonal migrant farmworkers to identify their health needs.^50^ Results identified service gaps in prenatal, vision, and hearing care, and a lack of Spanish‐language healthcare information for migrant workers.^50^ Another study addressed health disparities among Somali Bantu and Bhutanese child and adolescent refugees in Massachusetts. Again, language was cited as the main barrier (83%).^51^ Likewise, the lack of a common language was found in a qualitative paper from South Africa.^52^ Zimbabweans interpreted the language barrier as an imposition, exacerbated by their experiences outside of healthcare. They felt that no effort was made to link patients with healthcare providers which shared the same language (or second language) as them, and they felt discriminated against.^52^

In a similar vein, women in Canada valued providers who were culturally sensitive to issues of heteronormativity, ableism, and race, and they appreciated the opportunity to use trained and confidential interpreters or teletype communication in encounters with healthcare providers.^53^ However, a cross‐sectional study conducted in Norway investigating language barriers noted that professional language assistance remains underutilised in the health‐care sector.^54^ It was noted that there was a need to raise awareness about the legal responsibility healthcare providers have to ensure the sufficient level of communication with their patients/clients.^54^

An Australian study investigated the barriers and facilitators people with diabetes from a non‐English speaking background experienced when managing their medications.^55^ They found poor communication resulted in non‐adherence and, consequently, medication related problems.

A cohort study was conducted to identify barriers to participation as well as recruitment strategies to engage minority parents of young children in health research in Canada.^56^ The authors found direct contact between participants and research team members (e.g., during community events) as well as referrals by someone they trusted (e.g. a friend) were the most effective recruiting strategies.

There appears to be a difference in the barriers to participation in research as defined by community members themselves, and health professionals' perceptions of these barriers^57^ and that strong local research culture is needed to recruit participants into studies.^58^ Adequate time to discuss research projects was also seen as a way to increase recruitment of minority groups into studies.^59^

Congruence between the researcher and the participant aids satisfaction with research and recruiting healthcare workers and researchers skilled in Spanish and English may be more cost‐beneficial in Spanish speaking areas than providing translation services.^60^ Similarly, advertising in minority languages could boost recruitment into studies^61^ for example, Portuguese adverts on Facebook, etc.^61^ Research from the Netherlands^62^ and Sweden^63^ supports the stance that patients engage more with the research process if translators or translated materials are provided in different languages for different communities.^62^ Other researchers have noted that being able to opt to be interviewed and give verbal consent to participate in a language other than English, facilitated participation by lay community members in Australia.^64^ Also, researchers in Denmark suggested that survey response rate could increase with the use of different language versions of the national health survey.^65^

A SR aimed to identify barriers to providing healthcare services to ethnic minority patients from the perspective of nurses.^66^ The authors found that on‐site interpreters were not always available, and telephonic interpreters were unable to translate complex healthcare issues or to see facial expressions.^66^ A research synthesis noted that there is enough evidence of language barriers in health research by now, therefore future research should concentrate on the effectiveness and cost‐effectiveness of providing language concordant care.^67^

An oral health study in a Hispanic community found results similar to previous studies: Spanish language preference (lower acculturation) was associated with poor health related quality of life (HRQOL) and that culturally specific interventions aimed at improving oral health and preventing adverse consequences were needed.^68^

In Bangladesh, despite two languages being used in the medical context (English and Bengali) there are many minority languages that are not used in the medical context. The qualitative findings show that at least some respondents would have liked all their medical instructions in Bengali to achieve a successful treatment outcome.^69^

A study conducted in Australia to investigate the bilingual skills of practicing psychologists found most participants to be trained in English and expressed concerns about their application of psychological concepts in other languages, despite good conversational fluency.^70^ A similar finding was noted in a qualitative study from China: Cultural competency training and education provision should be provided for those caring for South Asian patients in Hong Kong.^2^ A case report by a graduate medical student provided real‐life examples regarding language and communication and noted that language concordant care providers and professional medical interpreters are invaluable for LEP individuals with limited education.^71^

A SR investigating language barriers in healthcare for Latino immigrants living with spina‐bifida found only seven articles out of 18 included Hispanics/Latinos.^72^ Making an effort to include minority groups in research is one way to address racial and ethnic health disparities.^73^

### 3.6 Theme 6: Racial and ethnic gap

The final theme included two papers, one reported on ‘Racial and ethnic gaps in healthcare’, and the other was ‘Racial and ethnic gaps in health research’. The most common strategy to improve minority health was delivering education and training (37%). The least common strategies were providing financial incentives (5.9%) and enhancing language and literacy services (0.4%).^74^ A qualitative study reported on racial and ethnic gaps in health research and found low literacy levels as the main barrier to ethnic minority recruitment and retention into neurological trials.^75^

## 4 DISCUSSION

The 74 papers included in this SR highlight that language barriers still exist in healthcare research ranging from not recognising ethnic minorities as part of the process of recruiting participants to not providing information in people's preferred language and including lack of satisfactory interpretation services in healthcare settings. This review has noted that during the past 40 years the focus on disparities in ethnic minority population research has been on the participant. The findings of this SR highlight the need to focus on methods of participant recruitment, recruitment of researchers, and workforce planning in health services to serve minority patients appropriately.^74^ This SR demonstrates that miscommunication between health providers and patients acts as a barrier to achieving an effective health service.^69^

Language barriers between researchers and participants present significant methodological challenges for researchers undertaking cross‐language qualitative studies.^44^, ^76^ Other barriers include cultural and research barriers,^44^, ^76^ for example, Tatari's paper on cancer screening^49^ showed that if the information sheet was not available in the participant's language, potential participants would disregard the study and opt not to partake. Similarly, Squires^76^ noted that for the rigour of a study improves if the investigators explain why they chose one language for the analysis in place of another.^76^

Authors have commented on cultural barriers to research and have tried to include aboriginal peoples in academic health research.^25^ Not considering the participants' cultural background violates their fundamental rights to ensure equitable representations in an already marginalised population.^25^ Extra efforts to recruit participants from ethnic minorities may be needed and comes with additional costs to the research, which should be considered within the planning stages.^31^ Partnerships between academic researchers and aboriginal peoples need to be fostered to improve trust and improve participation to address health related research questions.^30^

This SR presents evidence that therapeutic relationships and trust improves when culture is considered.^2^ Researchers frequently fail to include participants who experience language barriers in their projects, in part, because they lack the knowledge and experience to do so.^40^ There is a need to incorporate facilitators to recruitment by organising researcher training and resource allocation; so that this becomes a pre‐emptive measure to counteract barriers rather than a post‐event reflection on what the barriers were.^31^

Most data collection methods within social and medical sciences are developed with what is sometimes called the WEIRD sample, that is, people within Western Educated, Industrialised, Rich and Democratic societies.^77^ This gives way to one of the major concerns when doing research, which is whether the constructs that are being studied have the same meaning and value across cultures or even exist in all cultures.^41^ In terms of changes to policy and practice, authors have noted that there are issues that need to be considered, such as matching up researcher ethnicity with the ethnic group under study.^31^ Further efforts to improve the quality of research are needed to be useful for decision‐makers^29^

Researchers conducting studies including ethnic minorities should be cognisant of the customs, values, and beliefs of the target group(s) before designing any project. Issues of cross language data collection should be seen as a challenge and not as an obstacle, a stimulus to innovative thought and the development of new techniques of investigation. Cultural and linguistic differences are not always considered in health and care research or in health promotion. Individuals' reactions to illness and discomfort, their concepts of health, their help seeking behaviour is intimately bound up with cultural beliefs, values, and experience.^18^

Despite the historical lack of drive to close the knowledge translation gap between research and minority‐language communities within healthcare systems, durable inequalities still persist in healthcare around the world, including in Wales, where Welsh is a minority language spoken by 19% of the population.^78^ Healthcare organisations in Wales now have a statutory duty to deliver equitable Welsh language services.^4^ However, this SR did not yield any papers from Wales, indicating that there is still some way to go in Wales to bring the importance of the Welsh language into focus. In Wales, as in Canada,^79^ there has been a political momentum to deliver the ‘active offer’ in healthcare situations. In Canada, the active offer is relevant to the minority languages of English or French (dependent on region), and in Wales, the active offer relates to the use of Welsh as a minority language within a majority English speaking community. The active offer of Welsh is promoted as a policy by the Welsh Government^80^ and refers to the act of offering services in Welsh before a patient or client has to ask for it.

The evidence presented in this review paper suggests that there are still barriers to minority group representation in HSCR. There is general agreement that all the barriers have been recorded over the past few years and that now, the focus moving forward should be on increased effort to recruit minority language speakers, and record ethnicity in research participation to ensure transparent and robust research findings. We propose that future research should include details of ethnicity and languages spoken. Highlighting this may enable researchers to consider language and culture whilst interpreting the results and formulating the recommendations. Thereby ensuring that language and culture is considered throughout the whole research process.

Based on these results, we recommend that:

1. Ethnic background and languages spoken by the research participants should be identified and addressed throughout the research process (from design of the study to dissemination of findings).
2. Language and cultural preferences are appropriately considered/included in the analysis.

Adopting the same philosophy as the Tri‐Council Policy Statement or TCPS 2 (2014),^40^ the authors of this paper propose the use of an acronym as an aide memoir to help researchers to remember about including minority language speakers in HSCR, using the word.

RESEARCH:

R – Respond to the needs of minority language groups
E – Educate the research community to consider including minority language groups to ensure academic rigour
S – Reassure minority language communities that it is Safe to take part in research in HSCR
E – Ensure equity to all members of society
A – Aim to include minority language groups
R – Representative number of minority language groups
C – Consider and include minority language groups
H – Hear the voices of the representatives of the minority language groups

## CONFLICT OF INTEREST STATEMENT

The authors report no conflict of interest.

## ETHICS STATEMENT

Ethical approval is not required by our institute for carrying out Systematic Reviews of the literature.

## Supporting information

Supporting Information S1

Supporting Information S2

Supporting Information S3

## Data Availability

The data underlying this article will be shared on reasonable request to the corresponding author.
